# The α-arrestin SUP-13/ARRD-15 promotes isoform turnover of actin-interacting protein 1 in *Caenorhabditis elegans* striated muscle

**DOI:** 10.1093/pnasnexus/pgad330

**Published:** 2023-10-11

**Authors:** Mario Lewis, Kanako Ono, Zhaozhao Qin, Robert C Johnsen, David L Baillie, Shoichiro Ono

**Affiliations:** Department of Pathology, Emory University School of Medicine, Atlanta, GA 30322, USA; Department of Cell Biology, Emory University School of Medicine, Atlanta, GA 30322, USA; Department of Pathology, Emory University School of Medicine, Atlanta, GA 30322, USA; Department of Cell Biology, Emory University School of Medicine, Atlanta, GA 30322, USA; Department of Molecular Biology and Biochemistry, Simon Fraser University, Burnaby, BC V5A 1S6, Canada; Department of Molecular Biology and Biochemistry, Simon Fraser University, Burnaby, BC V5A 1S6, Canada; Department of Molecular Biology and Biochemistry, Simon Fraser University, Burnaby, BC V5A 1S6, Canada; Department of Pathology, Emory University School of Medicine, Atlanta, GA 30322, USA; Department of Cell Biology, Emory University School of Medicine, Atlanta, GA 30322, USA; Winship Cancer Institute, Emory University School of Medicine, Atlanta, GA 30322, USA

**Keywords:** actin, actin-interacting protein 1 (AIP1), α-arrestin, sarcomere, ubiquitination

## Abstract

Precise arrangement of actin, myosin, and other regulatory components in a sarcomeric pattern is critical for producing contractile forces in striated muscles. Actin-interacting protein 1 (AIP1), also known as WD-repeat protein 1 (WDR1), is one of essential factors that regulate sarcomeric assembly of actin filaments. In the nematode *Caenorhabditis elegans*, mutation in *unc-78*, encoding one of the two AIP1 isoforms, causes severe disorganization of sarcomeric actin filaments and near paralysis, but mutation in *sup-13* suppresses the *unc-78-*mutant phenotypes to restore nearly normal sarcomeric actin organization and worm motility. Here, we identified that *sup-13* is a nonsense allele of *arrd-15* encoding an α-arrestin. The *sup-13/arrd-15* mutation suppressed the phenotypes of *unc-78* null mutant but required *aipl-1* that encodes a second AIP1 isoform. *aipl-1* was normally expressed highly in embryos and downregulated in mature muscle. However, in the *sup-13/arrd-15* mutant, the AIPL-1 protein was maintained at high levels in adult muscle to compensate for the absence of the UNC-78 protein. The *sup-13/arrd-15* mutation caused accumulation of ubiquitinated AIPL-1 protein, suggesting that a normal function of *sup-13/arrd-15* is to enhance degradation of ubiquitinated AIPL-1, thereby promoting transition of AIP1 isoforms from AIPL-1 to UNC-78 in developing muscle. These results suggest that α-arrestin is a novel factor to promote isoform turnover by enhancing protein degradation.

**Significance Statement**

Sarcomeric organization of contractile proteins is essential for efficient contractility of striated muscles. However, the mechanism of sarcomere assembly remains unclear. Actin-interacting protein 1 (AIP1), also known as WDR1, is a conserved protein that promotes disassembly of actin filaments and is essential for sarcomeric assembly of actin filaments. In the nematode *Caenorhabditis elegans*, we found that the *sup-13/arrd-15* gene, encoding an α-arrestin, promotes turnover of one of the two AIP1 isoforms in striated muscle by specifically targeting one isoform for ubiquitin-dependent degradation. Our study suggests that α-arrestin is a novel factor to promote isoform transition in striated muscle.

## Introduction

Dynamic assembly and disassembly of actin filaments are critical for the function of the actin cytoskeleton that plays central roles in many cell biological processes including cell migration, cell division, and morphogenesis. Because actin filaments are relatively stable, severing and depolymerization of actin filaments are often promoted for cytoskeletal reorganization and recycling of actin monomers for new rounds of polymerization (1–3). Actin depolymerizing factor (ADF)/cofilin is a key regulator of actin filament disassembly by accelerating both depolymerization and severing of actin filaments. However, ADF/cofilin by itself can only modestly sever and depolymerize actin filaments. Therefore, ADF/cofilin cooperates with other factors including coronin, actin-interacting protein 1 (AIP1), and cyclase-associated protein to promote rapid actin filament disassembly.

AIP1 (4), also known as WD-repeat protein 1 (WDR1), is an evolutionarily conserved regulator of actin filament dynamics (5, 6). AIP1 contains two seven-bladed β-propeller domains in a single molecule (7, 8) and promotes disassembly of actin filaments in the presence of ADF/cofilin (9–11). AIP1 has minimum effects on bare actin filaments but rapidly and strongly severs ADF/cofilin-decorated actin filaments (12–18). Coronin promotes binding of ADF/cofilin to actin filaments and further accelerates filament disassembly by AIP1 (16, 18, 19). In contrast, tropomyosin prevents binding of ADF/cofilin to actin filaments and inhibits filament disassembly by AIP1 (20). However, other than the regulation at the level of ADF/cofilin, how AIP1-mediated actin filament disassembly is regulated remains unknown. Since coronin, ADF/cofilin, and AIP1 are present in most eukaryotic cells (2), cells need to be equipped with a mechanism to control these strong actin filament disassembly factors in order to maintain functional actin cytoskeleton.

Mutation or knockdown of AIP1 causes abnormal actin filament dynamics and organization in vivo in most tested organisms (5, 6). Notably, point mutations in the human AIP1 gene, *WDR1*, cause severe blood disorders, including reduction and impaired motility of neutrophils (21–23). Therefore, *WDR1* is considered a gene responsible for lazy leukocyte syndrome (24, 25). Similar *WDR1* mutant phenotypes are reported in mice (26) and Zebrafish (27). AIP1 is also an essential regulator of sarcomere assembly in striated muscle. In the nematode *Caenorhabditis elegans*, mutations in the AIP1 gene, *unc-78*, cause severe disorganization of sarcomeric actin filaments and reduced muscle contractility (28–30). A cardiomyocyte-specific *WDR1* knockout in mice causes similar actin disorganization and results in postnatal lethality with hypertrophic cardiomyopathy (31). Thus, genetic studies on AIP1 should provide insight in the basic in vivo function of AIP1 as well as pathogenesis of AIP1/WDR1-linked diseases.

In this study, we took advantage of *C. elegans* genetics to analyze an extragenic suppressor of *unc-78* encoding AIP1. The *sup-13* mutation was previously shown to suppress the motility defects of *unc-78* mutants, but the molecular identity of *sup-13* was uncharacterized (32, 33). We identified that *sup-13* is a nonsense allele of *arrd-15* encoding a member of the α-arrestins that are generally known as adaptors to mediate protein trafficking and degradation (34–36). We found that *sup-13/arrd-15* is a regulator of a second AIP1 gene, *aipl-1*, that is partially redundant with *unc-78* (37, 38). In wild-type muscle, UNC-78 is the predominant AIP1 isoform in adults. However, in *sup-13/arrd-15* mutant muscle, AIPL-1 is maintained at high levels, without significant effects on UNC-78 levels, in adult muscle to compensate for the absence of UNC-78. Ubiquitinated AIPL-1 is accumulated in *sup-13/arrd-15* mutants, suggesting that *sup-13/arrd-15* mediates a process for degradation of ubiquitinated AIPL-1. Thus, our study shows that an AIP1 isoform is targeted for degradation in an α-arrestin-dependent manner in vivo and suggests a novel mechanism of isoform turnover by degradation of a selective isoform(s) in differentiated cells.

## Results

### sup-13 mutation suppresses sarcomeric actin disorganization of unc-78 mutants

**Fig. 1.**
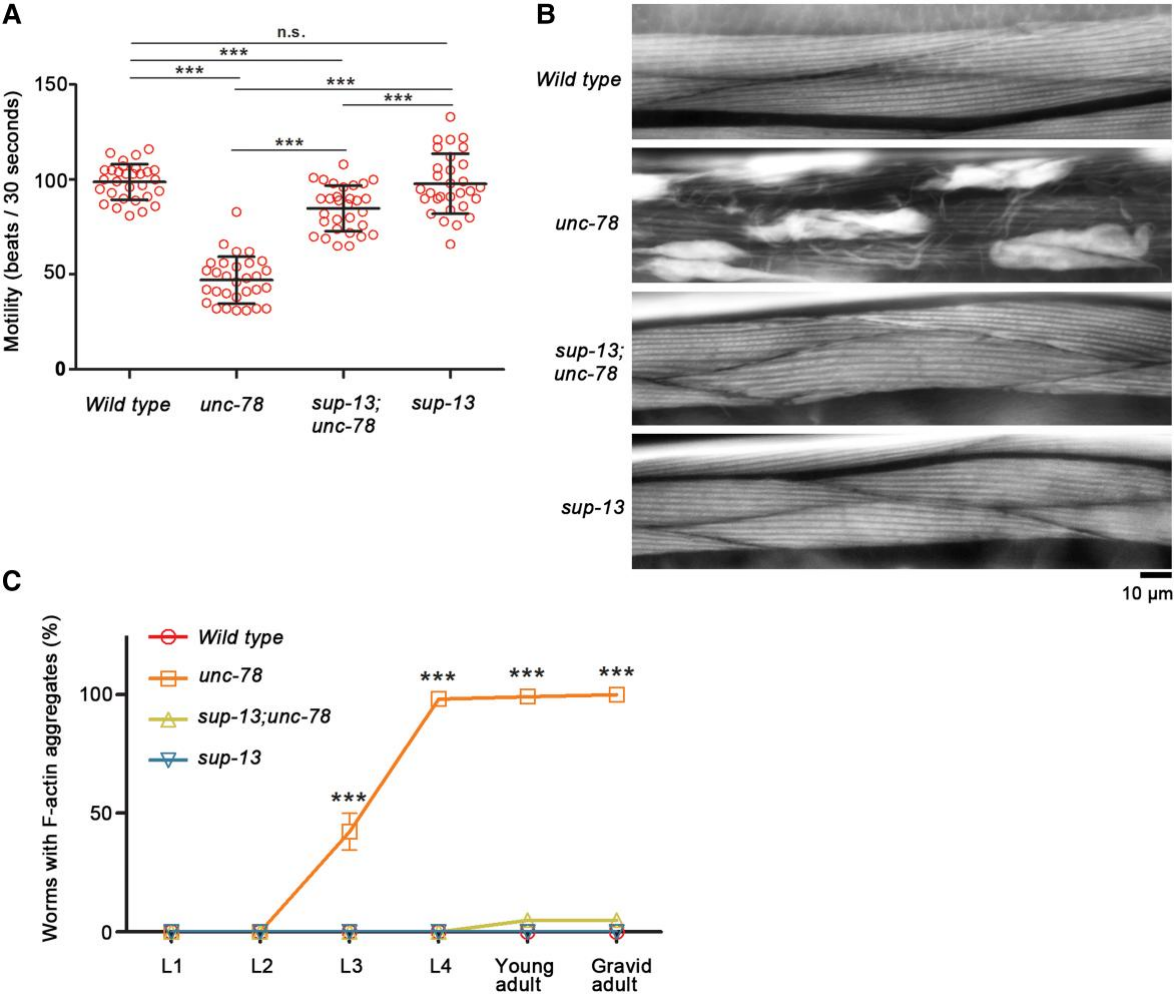
The *sup-13* mutation suppresses motility defects and sarcomere disorganization of the *unc-78* null mutant. A) Motility of young adult worms was quantified by measuring beating frequency (beats per 30 s). An *unc-78* null allele, *unc-78(gk27)*, was used in this study and simply shown as *unc-78* (*n* = 30, data are average ± standard deviation). ***, *P* < 0.001. n.s., not significant. B) Adult worms were stained for F-actin by tetramethylrhodamine-phalloidin, and regions of the body wall muscle are shown. Actin was organized in a striated manner in wild type but highly disorganized with aggregates in *unc-78(gk27)*. This phenotype was suppressed in the *sup-13(st210);unc-78(gk27)* double mutant to nearly an appearance of wild type. The *sup-13(st210)* single mutant did not show a detectable phenotype in the F-actin organization. Bar, 10 μm. C) Worms at different developmental stages were stained for F-actin by tetramethylrhodamine-phalloidin, and percentages of worms with F-actin aggregates (counted as positive when >25% of total muscle cells contained aggregates) were analyzed. For each experiment, > 500 worms were examined for each stage, and average ± standard deviation of three independent experiments are shown. ***, *P* < 0.001.

In an effort to identify genes with a functional connection to *unc-78*, a *sup-13* mutant allele, *st210*, was isolated from a screen for extragenic suppressors of *unc-78* and mapped to chromosome III (32, 33). The *unc-78* single mutants show sluggish and slow movement (28, 39), whereas the *sup-13;unc-78* double mutants show superficially normal motility (33). The original screen and analysis were performed using *unc-78(e1217)*, a loss-of-function allele with a missense mutation (28, 30). Therefore, we tested whether the *sup-13* mutation suppresses the phenotypes of *unc-78(gk27)*, a null allele expressing no detectable UNC-78 protein (28, 30). The *unc-78(gk27)* single mutants moved much slower than wild type (Fig. 1A). The *sup-13;unc-78(gk27)* double mutants moved slightly slower than wild type but significantly faster than the *unc-78(gk27)* single mutants (Fig. 1A). The *sup-13* single mutants moved as fast as wild type (Fig. 1A). Therefore, the *sup-13* mutation suppresses the motility defects of the *unc-78* mutants even in the absence of the UNC-78 protein.

The *sup-13* mutation also strongly suppressed disorganization of sarcomeric actin filaments in the body wall muscle of the *unc-78* mutants (Fig. 1B). The body wall muscle is responsible for body movement of the worms, and actin filaments were organized in a striated pattern in wild type (Fig. 1B). In the *unc-78(gk27)* mutants, actin filaments were highly disorganized and accumulated in large aggregates (Fig. 1B). However, in the *sup-13;unc-78(gk27)* double mutants, striated organization of actin filaments were nearly indistinguishable from that in wild type (Fig. 1B). The *sup-13* single mutants did not exhibit detectable defects in the actin filament organization (Fig. 1B). During development, the actin disorganization phenotype became detectable in *unc-78(gk27)* at the L3 larval stage and was nearly 100% penetrant after the L4 larval stage (Fig. 1C). The *sup-13* mutation almost completely suppressed the actin disorganization phenotype of *unc-78(gk27)* at all developmental stages (Fig. 1C), and the *sup-13* mutation alone did not cause actin disorganization at any stage (Fig. 1C). These cytological observations indicate that the *sup-13* mutation suppresses the muscle defects of the *unc-78* mutants by suppressing disorganization of sarcomeric actin filaments to restore assembly of functional contractile apparatuses.

### sup-13 is an allele of arrd-15 encoding an α-arrestin

**Fig. 2.**
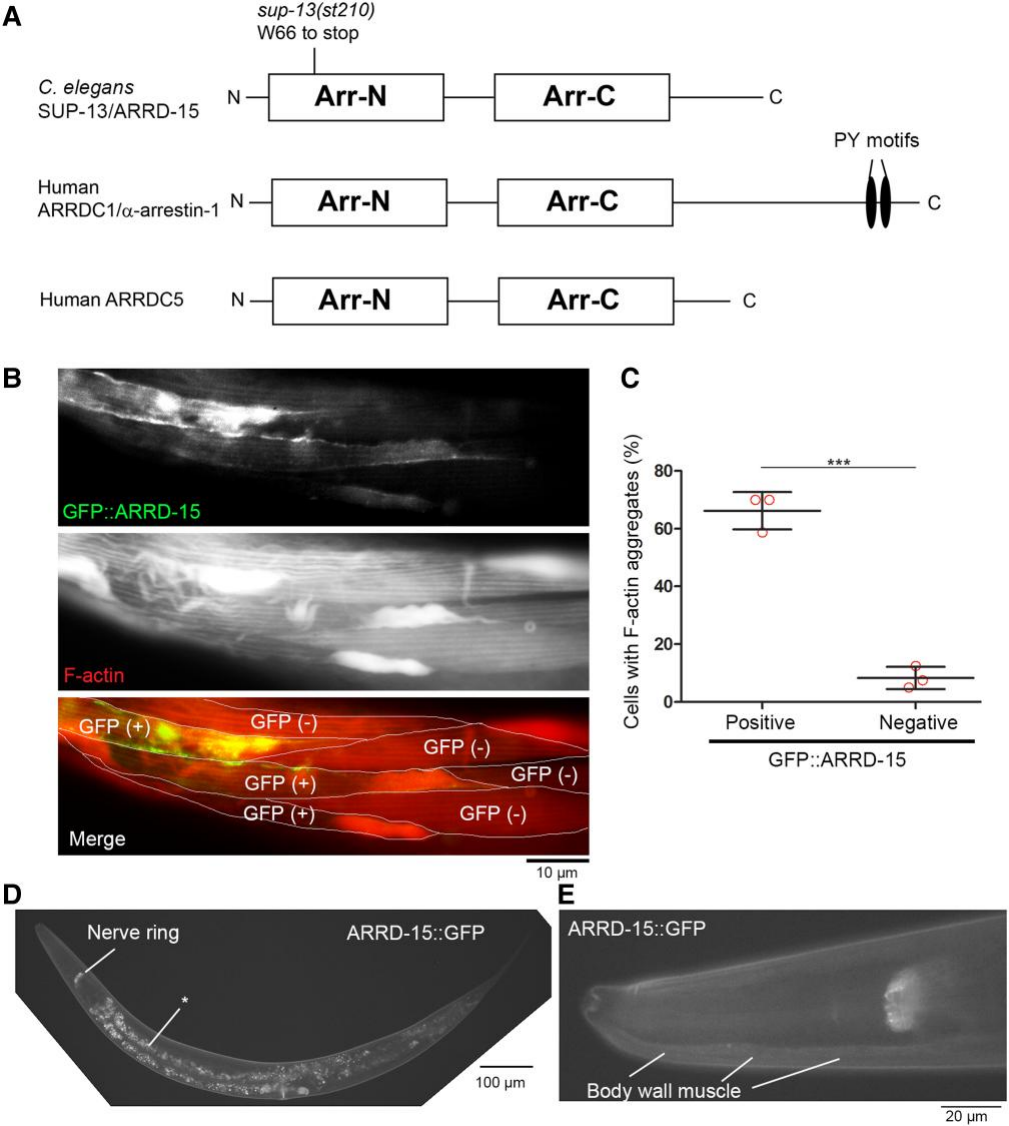
*sup-13* is a nonsense allele of *arrd-15* encoding an α-arrestin. A) Domain organization of the *C. elegans* SUP-13/ARRD-15 protein (374 amino acids, NCBI Reference Sequence: NP_001254970.1) with a location of the *sup-13(st210)* mutation. Arrestin-N (Arr-N) and arrestin-C (Arr-C) domains are indicated by boxes. N- and C-termini are shown as N and C, respectively. For comparison, human ARRDC1/α-arrestin 1 (433 amino acids, NCBI Reference Sequence: NP_689498.1) and ARRDC5 (342 amino acids, NCBI Reference Sequence: NP_001073992.1) are shown. Multiple copies of PY motifs (PP × Y) are present in the C-termini of many α-arrestins but absent in SUP-13/ARRD-15 and human ARRDC5. B, C) Transgenic rescue of the *sup-13* suppressor phenotype was demonstrated by expressing GFP::ARRD-15 in the body wall muscle of *sup-13(st210);unc-78(gk27)* using the *myo-3* promoter. The worms were stained with tetramethylrhodamine-phalloidin, and sarcomeric actin filaments (F-actin) were examined in GFP::ARRD-15 positive [GFP (+)] and negative [GFP (−)] cells. In the merged image (GFP::ARRD-15 in green and F-actin in red), cell-cell boundaries are marked by white borders. B). Bar, 10 μm. The presence or absence of F-actin aggregates was examined in GFP::ARRD-15 positive and negative cells (34–40 cells per experiment) from worms with mosaic expression of GFP::ARRD-15, and percentages of cells with F-actin aggregates are shown in the graph (C). Data are average ± standard deviation of three independent experiments. ***, *P* < 0.001. D, E) Expression pattern of SUP-13/ARRD-15 in adult worms was examined by tagging endogenous SUP-13/ARRD-15 with GFP. Fluorescence images at low (D; bar, 100 μm) and high (E; bar 20 μm) magnifications show the entire worm and the head region, respectively. SUP-13/ARRD-15::GFP was detected in the nerve ring (D) and body wall muscle in a diffuse pattern (E). Because the fluorescence intensity of SUP-13/ARRD-15::GFP was weak, autofluorescence of the gut granules (indicated by an asterisk in D) appears relatively high.

To understand the molecular nature of the *sup-13* mutation, we conducted whole-genome sequencing and identified that *sup-13(st210)* was a nonsense mutation [W66 (TGG) to stop (TGA)] in the *arrd-15* gene encoding an α-arrestin (Fig. 2A). The SUP-13/ARRD-15 protein (NCBI Reference Sequence: NP_001254970.1) is a previously uncharacterized member of α-arrestins, also known as arrestin-domain-containing proteins (ARRDCs) (Fig. 2A). These proteins commonly contain arrestin-N (Arr-N) and arrestin-C (Arr-C) domains (34–36) (Fig. 2A). In yeast, α-arrestins are also named arrestin-related trafficking adaptors (ARTs) and involved in protein trafficking and degradation as adaptors for ubiquitin (Ub) ligases (34–36). Many α-arrestins have multiple PY motifs (PP × Y or LP × Y) in their C-termini as binding sites for the WW domains of E3 Ub ligases (34–36) (Fig. 2A). However, SUP-13/ARRD-15 lacks a PY motif in the C-terminus, which is similar to mammalian ARRDC5, the least characterized member of α-arrestins (Fig. 2A).

The identity of *arrd-15* as *sup-13* was confirmed by transgenic rescue experiments. Transgenic expression of an N-terminally tagged green fluorescent protein (GFP)::ARRD-15 fusion protein in the body wall muscle of *sup-13(st210);unc-78(gk27)* suppressed the suppressor phenotype and induced actin aggregate formation (Fig. 2B). GFP::ARRD-15 was expressed in the body wall muscle of *sup-13(st210);unc-78(gk27)* using the *myo-3* promoter from extrachromosomal arrays, which often cause transgenic expression in a mosaic manner (40). The mosaic expression of GFP::ARRD-15 allowed us to compare GFP::ARRD-15-positive and negative cells within the same animals and populations. Nearly 70% of GFP::ARRD-15-positive cells had F-actin aggregates (Fig. 2B and C), whereas over 90% of GFP::ARRD-15-negative cells had striated actin organization with no aggregates (Fig. 2B and C), indicating that the suppression of the Sup-13 suppressor phenotype was caused by the transgenic expression of GFP::ARRD-15.

Expression of ARRD-15 in the body wall muscle was detected by observing localization of endogenous *sup-13/arrd-15* that was C-terminally tagged with GFP (ARRD-15::GFP) by CRISPR/Cas9-mediated gene editing (Fig. 2D and E). The fluorescence levels of ARRD-15::GFP were strongest in the nerve ring (Fig. 2D) and also detected at low levels in the body wall muscle and localized in the diffuse cytoplasm (Fig. 2E). This expression pattern is consistent with the mRNA distribution data in the GExplore database (41) (Online Supplementary Fig. S1). Together, these results of muscle-specific transgenic rescue and endogenous expression of *sup-13/arrd-15* in the body wall muscle indicate that *sup-13/arrd-15* acts autonomously in the body wall muscle to influence sarcomeric actin organization.

### The second AIP1 isoform, aipl-1, is required for sup-13/arrd-15-dependent phenotypic suppression of the unc-78 mutant phenotypes

**Fig. 3.**
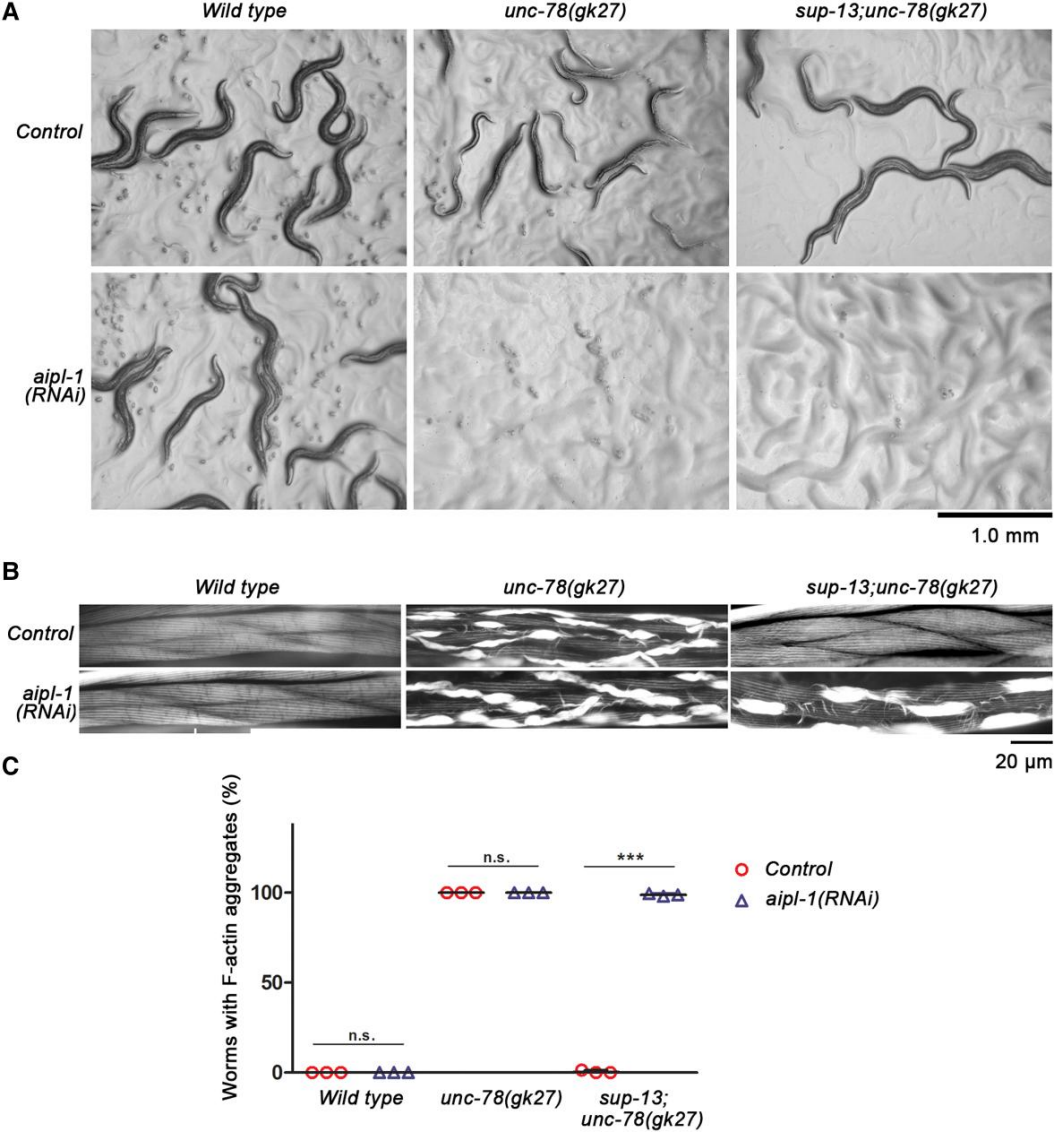
The second AIP1 gene *aipl-1* is required for *sup-13/arrd-15-*dependent suppression of the *unc-78* mutant phenotypes. A) Wild type, *unc-78*, or *sup-13;unc-78* worms were treated with control RNAi or *aipl-1(RNAi)* from the L4 larval stage, and the phenotypes were examined on agar plates in the next generation. In control RNAi, all strains grew to adult worms. However, in *aipl-1(RNAi)*, only wild type grew to adult worms, but *unc-78* and *sup-13;unc-78* were arrested as embryos. Bar, 1.0 mm. B) Wild type, *unc-78*, or *sup-13;unc-78* worms were treated with control RNAi or *aipl-1(RNAi)* from the L1 larval stage, and stained for F-actin with tetramethylrhodamine-phalloidin when they became adults, and regions of the body wall muscle are shown. In adult muscle, *aipl-1(RNAi)* did not affect the F-actin organization in wild type and *unc-78* but caused severe F-actin disorganization in *sup-13;unc-78*. Bar, 20 μm. C) Percentages of worms with F-actin aggregates (counted as positive when >25% of total muscle cells contained aggregates) were analyzed. For each experiment, >200 worms were examined for each sample, and average ± standard deviation of three independent experiments are shown (error bars are too small to be visible). ***, *P* < 0.001. n.s., not significant.

*Caenorhabditis elegans* has a second AIP1 gene, *aipl-1*, that is partially redundant with *unc-78* in actin regulation (37, 38). We found that *aipl-1* is essential for the phenotypic suppression by the *sup-13/arrd-15* mutation in the *unc-78* mutants (Fig. 3). Depletion of *aipl-1* in wild type by RNA interference (RNAi) did not cause a detectable abnormality (Fig. 3A, left). However, in the *unc-78(gk27)* mutants, *aipl-1(RNAi)* caused embryonic arrest (Fig. 3A, middle), as reported previously (37), and this lethal phenotype was not suppressed by the *sup-13* mutation (Fig. 3A, right). In order to observe adult phenotypes, the RNAi treatments were started from the L1 larval stage after normal embryogenesis was completed, and phenotypes analyzed in adult worms (Fig. 3B and C). Under these conditions, *aipl-1(RNAi)* did not cause detectable alterations in the phenotypes in wild type (Fig. 3B, left; Fig. 3C) and *unc-78(gk27)* (Fig. 3B, middle; Fig. 3C) but caused disorganization of actin filaments with aggregate formation in *sup-13;unc-78(gk27)* (Fig. 3B, right; Fig. 3C). These results and our previous reports demonstrate that *unc-78* and *aipl-1* have functional impacts at different developmental stages: *unc-78* mutations cause stronger phenotypes in the late larval and adult stages than in the embryonic and early larval stages (28) (Fig. 1C), whereas *aipl-1(RNAi)* (in an *unc-78* background) causes stronger phenotypes in the embryonic stages than in the adult stage (37). However, the strong adult phenotypes of *aipl-1(RNAi)* in *sup-13;unc-78(gk27)*, but not in *unc-78(gk27)*, suggest that *aipl-1* became functionally significant at the adult stage in the *sup-13* mutant background.

### sup-13/arrd-15 controls the protein level of AIPL-1, but not UNC-78, possibly in a Ub-dependent manner

**Fig. 4.**
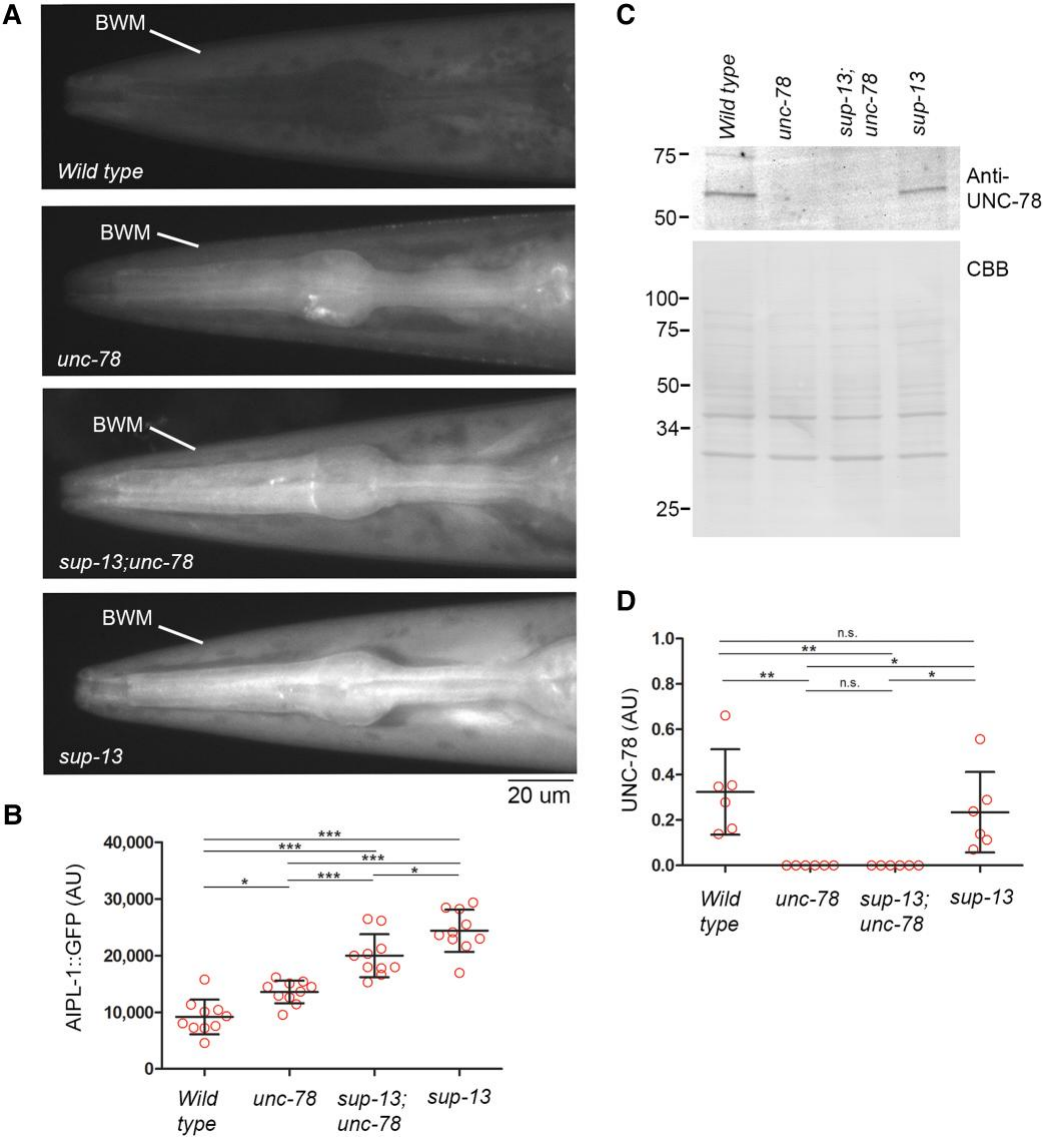
The *sup-13/arrd-15* mutation increases the protein levels of AIPL-1 in the body wall muscle. A, B) The AIPL-1::GFP protein levels in the body wall muscle were determined in the head region where the autofluorescence of the gut granules is absent. Fluorescence images of AIPL-1::GFP were captured using the same exposure settings for the four strains (A; bar, 20 μm), and the AIPL-1::GFP protein levels were determined as fluorescence intensity (arbitrary units) within the body wall muscle (BWM in A) in B. Data are average ± standard deviation (*n* = 10). *, 0.01 < *P* < 0.05. ***, *P* < 0.001. C, D) The UNC-78 protein levels in whole worm lysates were determined by Western blot using anti-UNC-78 antibody. Whole worm lysates of an equal number of young adults (30 worms) were loaded for each strain and probed with anti-UNC-78 antibody (C, top). Total proteins were stained with Coomassie Brilliant Blue (C, bottom) and used as a loading control to normalize the UNC-78 signals (arbitrary units) in D. Positions of molecular weight markers in kDa are shown on the left. Data are average ± standard deviation (*n* = 6). *, 0.01 < *P* < 0.05. **, 0.001 < *P* < 0.01. n.s., not significant.

Examination of AIPL-1 protein levels revealed that the *sup-13/arrd-15* mutation altered the AIPL-1 protein level in the adult body wall muscle (Fig. 4). Since AIPL-1 is expressed in many tissues (37), the endogenous *aipl-1* gene was C-terminally tagged with GFP by CRISPR/Cas9-mediated gene editing, and its expression in the body wall muscle was examined by fluorescence microscopy. The AIPL-1::GFP protein levels were determined as fluorescence intensity. In wild type, AIPL-1::GFP was very low in the body wall muscle (Fig. 4A and B) and slightly increased in *unc-78(gk27)* (Fig. 4A and B). However, in *sup-13;unc-78(gk27)* and *sup-13*, AIPL-1::GFP was significantly increased in the body wall muscle as compared with wild type and *unc-78(gk27)* (Fig. 4A and B), indicating that the *sup-13/arrd-15* mutation was responsible for the high levels of AIPL-1::GFP in the body wall muscle. In contrast, the UNC-78 protein levels were not altered by the *sup-13/arrd-15* mutation (Fig. 4C and D). The UNC-78 protein was examined by Western blot using whole adult worm lysates because UNC-78 is predominantly expressed in the body wall muscle in adults (28, 30). Indistinguishable levels of UNC-78 were detected in wild type and *sup-13*, and the absence of UNC-78 was confirmed in *unc-78(gk27)* and *sup-13;unc-78(gk27)* (Fig. 4C and D). These results indicate that the *sup-13/arrd-15* mutation specifically increases the level of AIPL-1, but not UNC-78, in the adult body wall muscle. Therefore, a normal function of *sup-13/arrd-15* is to decrease the level of AIPL-1 in the adult muscle.

**Fig. 5.**
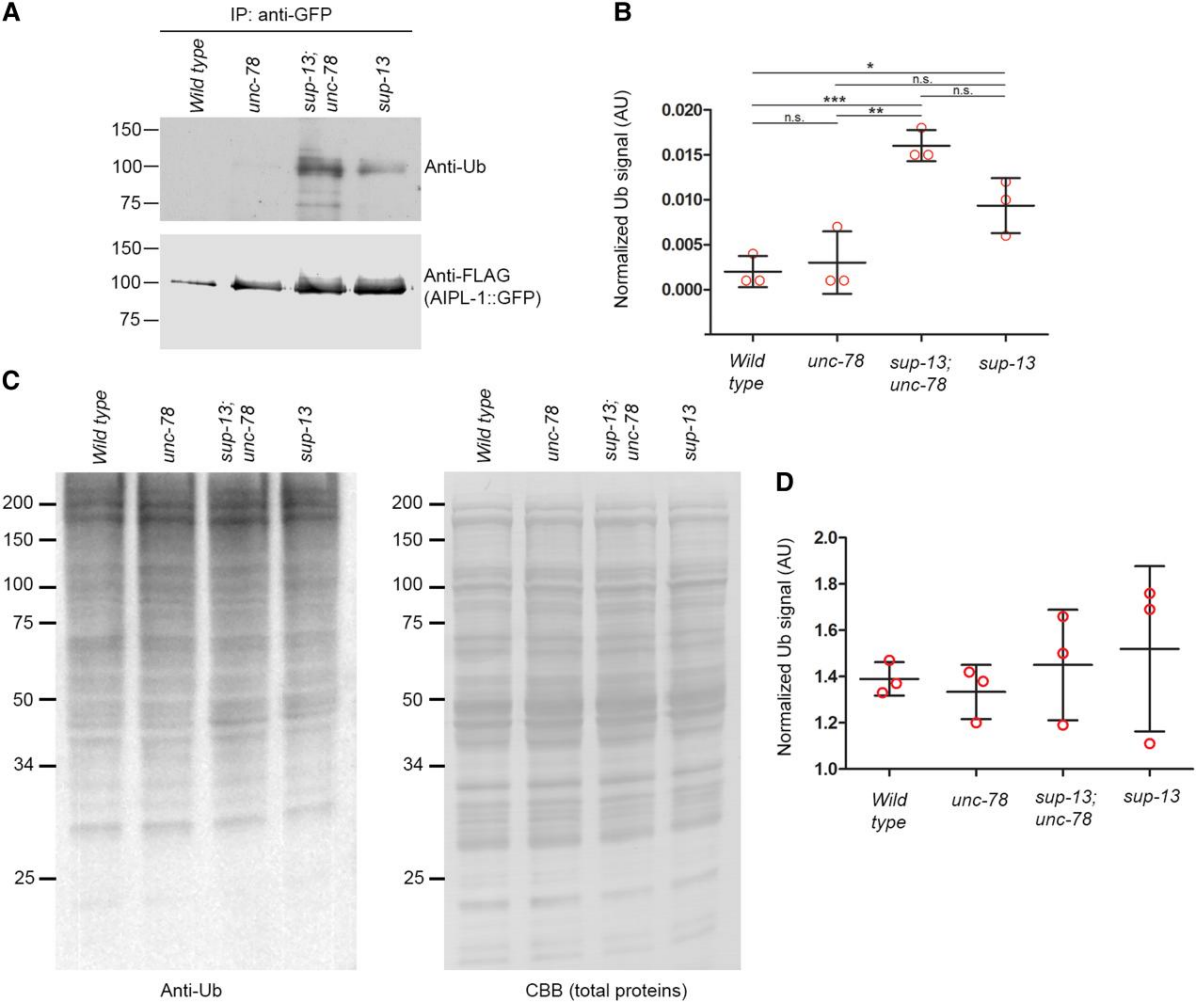
Ubiquitinated AIPL-1 is accumulated in the *sup-13/arrd-15* mutants. A, B) AIPL-1::GFP was immunoprecipitated with anti-GFP magnetic beads and probed with anti-Ub (Ub) antibody (A, top) and anti-DYKDDDDK (FLAG epitopes in AIPL-1::GFP) antibody (A, bottom). The anti-Ub signals (arbitrary units) were normalized to anti-DYKDDDDK signals and shown in the graph (B). Data are average ± standard deviation (*n* = 3). *, 0.01 < *P* < 0.05. **, 0.001 < *P* < 0.01. ***, *P* < 0.001. n.s., not significant. C, D) Whole lysates of adult worms were examined by Western blot using anti-Ub antibody (C, left). Total proteins on the blot were stained with Coomassie Brilliant Blue (CBB) (C, right) and used as a loading control to normalize the anti-Ub signals (arbitrary units) in D. Positions of molecular weight markers in kDa are shown on the left in A and C. Data are average ± standard deviation (*n* = 3). There were no significant differences among the data.

To determine the regulatory mechanism of AIPL-1 protein levels by *sup-13/arrd-15*, we examined the ubiquitination levels of AIPL-1 and found that ubiquitinated AIPL-1 was accumulated in the *sup-13/arrd-15* mutants (Fig. 5A and B). AIPL-1::GFP was immunoprecipitated, and probed with anti-Ub antibody in Western blot (Fig. 5A). Ubiquitinated AIPL-1::GFP was nearly undetectable in wild type and *unc-78(gk27)* but detected at high levels in *sup-13* and *sup-13;unc-78(gk27)* (Fig. 5A and B). Therefore, in the *sup-13/arrd-15* mutants, AIPL-1 protein might be maintained at high levels because ubiquitinated AIPL-1 was accumulated without being processed by the downstream protein degradation pathway. However, levels of ubiquitinated proteins in total worm lysates were not significantly different among the four strains (Fig. 5C and D), suggesting that *sup-13/arrd-15* regulates degradation of only specific target proteins rather than systemic degradation of ubiquitinated proteins. These results suggest that *sup-13/arrd-15* acts after the ubiquitination process and promotes degradation of ubiquitinated AIPL-1.

## Discussion

**Fig. 6.**
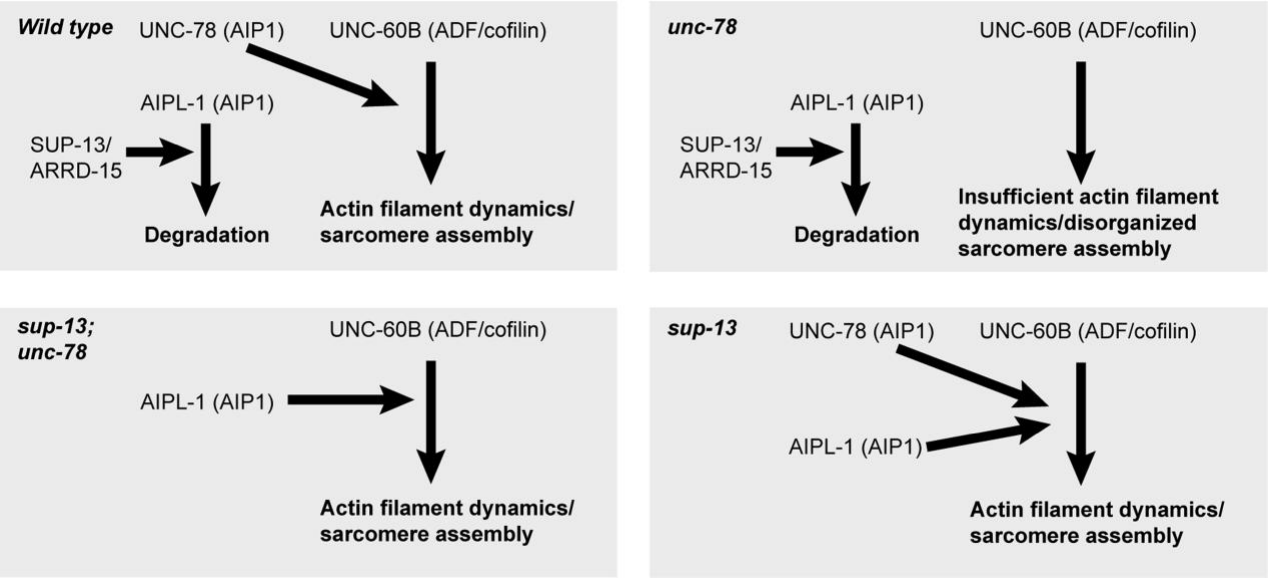
Model for the regulatory mechanism of AIP1 isoform turnover in the body wall muscle. The current study suggests that SUP-13/ARRD-15 specifically targets AIPL-1 for degradation in the body wall muscle. Therefore, in wild type, UNC-78 is the predominant AIP1 isoform that cooperates with UNC-60B (ADF/cofilin) to promote actin filament dynamics. In *unc-78* single mutants, there is not enough AIP1 proteins to promote actin filament dynamics, causing sarcomere defects. However, in *sup-13;unc-78* double mutants, AIPL-1 is not degraded and compensates for the absence of UNC-78. In *sup-13* single mutants, both UNC-78 and AIPL-1 promotes actin filament dynamics and do not cause apparent sarcomere defects.

In this study, we identified the molecular nature of the *sup-13/arrd-15* gene and characterized how the *sup-13/arrd-15* mutation suppresses the *unc-78* mutant phenotypes. Based on the current and previous data, we propose that the isoform turnover of AIP1 occurs at two levels in *C. elegans* muscle. First, expression of *unc-78* and *aipl-1* are transcriptionally regulated in an opposite manner as determined by the promoter analyses: the *unc-78* promoter is more active in adult muscle than embryonic muscle (30), whereas the *aipl-1* promoter is more active in embryonic muscle than in adult muscle (37). Therefore, during muscle development, expression of *unc-78* is increased, but that of *aipl-1* decreased. Second, the AIPL-1 protein is specifically cleared in adult muscle by *sup-13/arrd-15* in a Ub-dependent manner. In the *sup-13/arrd-15* mutants, AIPL-1 protein is maintained at high levels in adult muscle and is capable of compensating for the absence of UNC-78 in the *unc-78-*null mutants (Fig. 6). This is supported by our previous observations that forced expression of GFP::AIPL-1 in adult muscle of the *unc-78-*null mutants rescues the sarcomeric actin organization (37), indicating that AIPL-1 is sufficient for suppressing the *unc-78*-mutant phenotype. In addition, knockdown of *aipl-1* in *sup-13;unc-78(gk27)* suppressed the Sup-13 suppressor phenotype (Fig. 3), indicating that *aipl-1* is necessary for suppressing the *unc-78*-mutant phenotype in the *sup-13/arrd-15* mutant background. Thus, the transition of AIP1 isoforms in *C. elegans* muscle appears to be a highly regulated process.

Multiple AIP1 isoforms are present only in a limited number of species, and protein levels of AIP1 may be regulated in a species-specific manner. In *Arabidopsis thaliana*, two AIP1 isoforms are differentially expressed in reproductive and vegetative tissues and regulate actin organization in respective tissues (42–44). However, most species have only one gene encoding AIP1 (5, 6, 45). Mammals have a single AIP1 gene, known as *WDR1* (46). In the mouse heart, the WDR1 protein level is high in embryos and then gradually decreased by unknown mechanism during postembryonic development (31). AIP1 can strongly sever ADF/cofilin-bound actin filaments at very low concentrations in vitro (12–16). Therefore, high AIP1/WDR1 levels may be maintained when actin cytoskeletal reorganization is highly active in embryonic heart, but AIP1/WDR1 may be decreased when mature myofibrils are assembled and stabilized in postembryonic heart. However, it is still not clear why turnover of the AIP1 isoforms occurs in *C. elegans* muscle. Biochemically, UNC-78 and AIPL-1 are qualitatively very similar in promoting disassembly of ADF/cofilin-bound actin filaments (37), preference for ADF/cofilin isoforms (29, 37), and pH sensitivity (45). Genetically, *unc-78* and *aipl-1* are partially redundant in assembly of contractile apparatuses in striated and nonstriated muscles (37, 38). Therefore, there may be unknown differences in the function and/or regulation of the two AIP1 isoforms, which might be beneficial for *C. elegans*.

Generally, α-arrestins are known to promote ubiquitination of target proteins by recruiting E3 Ub ligases to the substrates (34–36). However, in our study, the AIPL-1 protein levels were increased with accumulation of ubiquitinated AIPL-1 protein in the *sup-13/arrd-15* mutants. These results suggest that SUP-13/ARRD-15 is involved in processing ubiquitinated AIPL-1 for degradation rather than promoting ubiquitination of AIPL-1. Alternatively, SUP-13/ARRD-15 may indirectly regulate degradation of ubiquitinated AIPL-1. If there is a negative regulator of AIPL-1 degradation, promoting Ub-dependent degradation of this regulator by SUP-13/ARRD-15 can accelerate AIPL-1 degradation. Therefore, additional investigation is needed to identify other factors involved in the SUP-13/ARRD-15-mediated regulation of AIPL-1.

Most of the previously characterized α-arrestins have multiple PY motifs (PP × Y or LP × Y) in their C-termini as binding sites for the WW domains of Rsp5/Nedd4-like E3 Ub ligases (34–36). However, SUP-13/ARRD-15 lacks this motif (Fig. 2A), and how it is involved in the degradation of a target protein is currently unknown. In mammals, arrestin-domain-containing 5 (ARRDC5) is the only α-arrestin lacking a PY motif, and its biological function is poorly understood. One recent study has shown that ARRDC5 is enriched in testis and required for normal spermatogenesis in mice (47), although whether ARRDC5 is involved in protein degradation is unknown. *C. elegans* has one β-arrestin gene, *arr-1* (48), and 28 α-arrestin genes. The *arr-1* β-arrestin gene is involved in G-protein-coupled receptor signaling (49). However, only two α-arrestin genes, *cnp-1/arrd-17* and *ttm-2*, have been previously characterized in *C. elegans*. *cnp-1/arrd-17* is required for chemosensation (50). *ttm-2* is required for defense against bacterial toxins (51). Either *cnp-1/arrd-17* or *ttm-2* does not have a consensus PY motif and has not been implicated in protein degradation. Therefore, metazoan α-arrestins lacking a PY motif may recruit a Ub ligase using an alternative mechanism such as phosphor-Ser/Thr dependent binding to a WW domain (52) or be involved in unknown biological processes.

Our study suggests that an α-arrestin is involved in isoform switching of muscle proteins during development. Many sarcomeric proteins undergo isoform transition during muscle differentiation, development, and under pathological conditions (53). Although isoform switching at levels of transcription and alternative splicing has been extensively studied, the clearance mechanism for old isoforms is still poorly understood. Since misexpression of inappropriate isoforms can cause pathological conditions in muscle, clearing old or misexpressed isoforms will be important to complete normal isoform transition. For example, abnormal expression of troponin T isoforms due to misregulated alternative splicing is implicated in the pathogenesis of myotonic dystrophy (54). Expression of a nonmuscle tropomyosin isoform in skeletal muscle can cause dystrophic phenotypes (55, 56). In addition to these structural proteins, cofilin isoforms also transition from a nonmuscle isoform (cofilin-1) to a muscle isoform (cofilin-2) during muscle differentiation (57, 58). Cofilin-1 is normally cleared by the Ub-proteasomal system after muscle differentiation, but failure to clear cofilin-1 from mature muscle causes sarcomere disorganization and impaired force generation thereby enhancing phenotypes in Emery-Dreifuss muscular dystrophy (59). The Ub-proteasomal system has been shown to be important for normal sarcomere assembly (60–62), and muscle atrophy, and hypertrophy (63–65). To date, Ub ligases, MuRF-1, MAFbx-1/atrogin-1, and Cbl-b have been associated with muscle atrophy (66–68), and UBR4 with hypertrophy (64), but function of an α-arrestin in a Ub-dependent pathway has not been determined in vertebrate muscle. Therefore, our study suggests a novel role of an α-arrestin in protein turnover in normal muscle development or under pathological conditions.

## Materials and methods

### *Caenorhabditis elegans* strains and culture

The worms were cultured following standard methods (69). N2 wild type, RW2337 *sup-13(st210);unc-78(e1217)*, and CB1217 *unc-78(e1217)* were obtained from the *Caenorhabditis* Genetics Center (Minneapolis, MN). ON5 *unc-78(gk27)* was reported previously (28). ON320 *sup-13(st210);unc-78(gk27)* was generated by crossing RW2337 and ON5 *unc-78(gk27)* and selecting *sup-13(st210);unc-78(gk27)* homozygotes in the F2 generation. ON341 *sup-13(st210)* was generated by crossing ON320 and N2 and selecting *sup-13(st210)* homozygotes in the F2 generation. Other transgenic strains are described below. All strains used in this study are listed in Online Supplementary Table S1.

### Motility assays

Worm motility was quantified as described previously (70). Briefly, adult worms were placed in M9 buffer. Then, one beat was counted when a worm swung its head to either right or left. The total number of beats in 30 s was recorded.

### Genomic DNA isolation and whole-genome sequencing

We sequenced RW2337 *sup-13(st210) III;unc-78(e1217) X* and CB1217 *unc-78(e1217) X* as described previously (71). Genomic DNA was submitted to the British Columbia Cancer Agency Canada’s Michael Smith Genome Sciences Centre for whole-genome sequencing using the Illumina PET HiSeq technology to produce 150 bp long paired end reads. Burrows-Wheeler Alignment (BWA) (72) was used to align the sequencing reads against the *C. elegans* reference genome (version WS249). SAMtools (73) was applied to remove duplicate reads. Integrative Genomics Viewer (74, 75) was employed to identify the breakpoints of large deletions, medium sized insertions, and translocations. Finally, CooVar (76) was used to examine the effect of the variations on the coding sequences. *sup-13* was previously mapped near the center of chromosome III by three-factor mapping using *dpy-17* and *lon-1* (32, 77). The sequences of RW2337 and CB1217 were compared, and a G > A mutation at III:8934387 was the only nonsense mutation (W66 to stop in *arrd-15*) within a 5-Mb interval in a protein-coding gene in the *sup-13* mutant (RW2337), which is an expected mutation frequency for ethyl methanesulfonate mutagenesis in *C. elegans* as reported in the Million Mutation Project (78). To determine whether this change is the *sup-13* mutation, RW2337 was outcrossed with ON5 *unc-78(gk27)*, and mutations in the F2 *sup-13;unc-27(gk27)* double homozygotes were analyzed. As a result, the nonsense mutation in the *arrd-15* gene was linked with the Sup-13 suppressor phenotype.

### Transgenic expression of GFP::ARRD-15 in the body wall muscle

The cDNA for ARRD-15 (NCBI Reference Sequence: NP_001254970.1) was cloned in-frame at the 3′ end of the GFP sequence of pPD-118.20 (provided by Dr. Andrew Fire, Stanford University) containing the *myo-3* promoter that is active in the body wall muscle (79), and injected into the syncytial gonad of ON320 *sup-13(st210) III;unc-78(gk27) X* to generate transgenic animals with extrachromosomal arrays as described (30). The generated strain is ON369 *sup-13(st210) III;unc-78(gk27) X;ktEx252[myo-3p::gfp::ARRD-15a, pRF4]*.

### CRISPR/Cas9-mediated genome editing

For the *aipl-1::gfp* strain, the GFP sequence was inserted in the genome in-frame at the 3′-end of the *aipl-1* coding region by CRISPR/Cas9-mediated genome editing using vectors and protocols described by Dickinson et al. (80). A single guide (sg) RNA target sequence (TATAAACATGAAAACTAGAG) was cloned into pDD162 (Addgene Plasmid # 47549) for expression of both sgRNA and Cas9 nuclease. Homology arms of ∼500 bp each were fused with the GFP::self-excising cassette region (also containing three copies of FLAG/DYKDDDDK epitopes) from pDD282 (Addgene Plasmid #66823) by fusion PCR using Q5 High-Fidelity DNA Polymerase (New England Biolabs) and used as a homologous repair template as described previously (81). Hygromycin-resistant worms with a roller phenotype were isolated as knock-in worms. They were treated at 34°C for 4 h to induce excision of the self-excising cassette (80). In the next generation, nonroller GFP-positive worms were isolated and outcrossed three times, and ON355 *aipl-1(kt4[aipl-1::3xFLAG::gfp])* was established.

For the *arrd-15::gfp* strain, the GFP sequence was inserted in the genome in-frame at the 3′-end of the *arrd-15* coding region by CRISPR/Cas9-mediated genome editing at SunyBiotech (Fujian, China), and PHX4137 *arrd-15(syb4137[arrd-15::gfp])* was isolated.

### RNAi experiments

RNAi experiments were performed by feeding with *Escherichia coli* expressing double-stranded RNA as described previously (82). An RNAi clone for *aipl-1* (V-9L16) was obtained from MRC Geneservice (Cambridge, United Kingdom) (83). An empty vector L4440 (provided by Dr. Andrew Fire, Stanford University) was used as a control. When L4 larvae were treated with RNAi, phenotypes were characterized in the F1 generation (82). When L1 larvae were treated with RNAi, phenotypes were characterized when the treated worms became adults (84).

### Immunoprecipitation of AIPL-1::GFP

Age-synchronized young adults were mixed with ice cold immunoprecipitation buffer (25 mM Tris pH 7.5, 150 mM NaCl, 1 mM EDTA, 5% glycerol, 0.5% IGEPAL CA-630, 1 mM phenylmethylsulfonyl fluoride, 1 μM leupeptin and 1 μM pepstatin) and mechanically lysed on ice with 20 passages through a Balch cell homogenizer with a 12 μm tungsten carbide ball bearing (Isobiotec, Heidelberg Germany) (85). The lysates were centrifuged at 16,100 × *g* for 10 min at 4°C. Protein concentrations were determined using a Pierce BCA Protein Assay Kit (catalog # 23225, Thermo Fisher Scientific, Waltham, MA), and an equal amount of proteins (> 1 mg) was used for immunoprecipitation. The lysates were incubated for 1 h at 4°C with 10 μl suspension (binding capacity for 0.8–1.1 μg of GFP) of GFP-Trap Magnetic Particles M-270 (catalog # gtd, ChromoTek, Planegg Germany), which was pre-equilibrated with the immunoprecipitation buffer. The magnetic particles were washed three times with the immunoprecipitation buffer, and bound proteins were eluted by adding 15 μl of sodium dodecyl sulfate (SDS) lysis buffer (2% SDS, 80 mM Tris-HCl, 5% β-mercaptoethanol, 15% glycerol, 0.05% bromophenol blue, pH 6.8) and heating at 95°C for 5 min. Magnetic particles were separated from solution and the supernatant stored at −20°C for downstream application.

### Western blotting

Whole worm lysates were prepared from an equal number of young adults (30 worms for Fig. 4C) by directly harvesting worms in 15 μl of SDS lysis buffer or harvesting and washing age-synchronized worms in M9 buffer (Fig. 5C) and lysis in SDS lysis buffer, which were heated at 95°C for 2 min followed by sonication, and another heating at 95°C for 2 min. Protein concentrations of the latter were determined by a filter-paper dye binding assay using Coomassie Brilliant Blue G (86), and 10 μg proteins were used for each sample. The lysates were subjected to SDS-polyacrylamide gel electrophoresis (SDS-PAGE) (10 or 12% polyacrylamide gel), and the proteins were transferred to a polyvinylidene fluoride (PVDF) membrane. The membranes were blocked with Bullet Blocking One (catalog # 13779-56, Nacalai USA, San Diego, CA) for 5 min at room temperature. For detection of UNC-78 (Fig. 4C), the blots were probed with rabbit anti-UNC-78 polyclonal antibody (1:500 dilution) (29), diluted in Signal Enhancer HIKARI solution (Nacalai USA, San Diego, CA). After washing with phosphate-buffered saline containing 0.1% Tween 20 (PBS-T), the membranes were treated with IRDye 680RD goat anti-rabbit IgG polyclonal secondary antibody (1:7,500 dilution) (catalog # 926-68071, Li-Cor, Lincoln, NE), washed with PBS-T, and scanned with the Odyssey Classic Infrared Imaging System (Li-Cor). For detection of ubiquitinated proteins in whole worm lysates (Fig. 5C), the blots were probed with anti-Ub mouse monoclonal (P4D1) antibody (1:1,000 dilution) (Cat # 3739, Cell Signaling, Danvers, MA), followed by horseradish peroxidase-conjugated goat anti-mouse IgG antibody (1:2,000 dilution) (catalog # 31431, Thermo Fisher Scientific, Waltham, MA). The signals were detected by incubating with a SuperSignal West Pico Chemiluminescent Substrate (catalog # 34080, Thermo Fisher Scientific), and images acquired by an Azure Biosystems c600 imaging system. The band intensity was quantified using Image Studio Lite (Li-Cor, NE USA) for Fig. 4C or ImageJ (87) for Fig. 5C, and normalized to total protein amounts as determined by staining membranes with Coomassie Brilliant Blue R-250 and densitometry using ImageJ as described previously (88).

To examine AIPL-1 ubiquitination in the immunoprecipitated samples, the samples were subjected to SDS-PAGE and transferring to PVDF membranes, as described above. The membranes were probed with anti-Ub mouse monoclonal (P4D1) antibody (1:1,000 dilution) (Cat # 3739, Cell Signaling, Massachusetts USA), followed by horseradish peroxidase-conjugated goat anti-mouse IgG antibody (1:2,000 dilution) (catalog # 31431, Thermo Fisher Scientific, Waltham, MA). The signals were detected by incubating with a SuperSignal West Pico Chemiluminescent Substrate (catalog # 34080, Thermo Fisher Scientific) and exposing to X-ray films. The membranes were blocked again, and probed with rabbit anti-DYKDDDDK epitope polyclonal antibody (1:5,000 dilution) to detect AIPL-1::GFP with three copies of the FLAG epitope (DYKDDDDK), followed by incubation with IRDye 680RD goat anti-rabbit IgG polyclonal secondary antibody (1:7500). After washing with PBS-T, the signals were detected by scanning with an Odyssey Classic Infrared Imaging System. The signals for anti-Ub and anti-DYKDDDDK were quantified with ImageJ and Image Studio Lite, respectively, and the anti-Ub signals were normalized to the anti-DYKDDDDK signals.

### Fluorescence microscopy

Staining of worms with tetramethylrhodamine-phalloidin (catalog # P1951, MilliporeSigma, St. Louis, MO) was performed as described (28, 89). Samples were observed by epifluorescence using a Nikon Eclipse TE2000 inverted microscope (Nikon Instruments, Tokyo, Japan) with a CFI Plan Fluor ELWD 40 × (NA 0.60) objective. Images were captured by a Hamamatsu ORCA Flash 4.0 LT sCMOS camera (Hamamatsu Photonics, Shizuoka, Japan) and processed by NIS-Elements AR V5.02.01 (Nikon Instruments) and Adobe Photoshop CS3.

To measure fluorescence intensity of AIPL-1::GFP, live worms were mounted and immobilized with 25% Pluronic F-127 (catalog # 2730-50G, Biovision, Milpitas, CA) in M9 buffer containing 0.5 mM levamisole and 0.1% tricaine methanesulfonate. Fluorescence images were captured with the same settings for all samples in NIS-Elements using a Nikon Eclipse TE2000 inverted microscope with a CFI Plan Fluor ELWD 40 × (NA 0.60) objective. Fluorescence intensity of each worm was determined using ImageJ as average fluorescence intensity at 10 randomly selected points within the cytoplasm of the body wall muscle in the head region.

### Statistics

The following methods were used for statistical tests in GraphPad Prism 5 (Version 5.04): one-way analysis of variance (ANOVA) and Tukey post hoc test (Figs. 1A; 4B and D; and 5B and D); two-way ANOVA and Bonferonni post hoc test (Fig. 1C); unpaired *t*-test (Figs. 2C and 3C).

## Supplementary Material

pgad330_Supplementary_Data

## Data Availability

All data are contained in the article.
