# Supplementary material for: The α-arrestin SUP-13/ARRD-15 promotes isoform turnover of actin-interacting protein 1 in Caenorhabditis elegans striated muscle
Source: PNAS Nexus. 2023 Oct 11;2(10):pgad330. doi: 10.1093/pnasnexus/pgad330 (PMC10590129; doi:10.1093/pnasnexus/pgad330)
Supplement: pgad330_Supplementary_Data [file pgad330_supplementary_data.pdf]

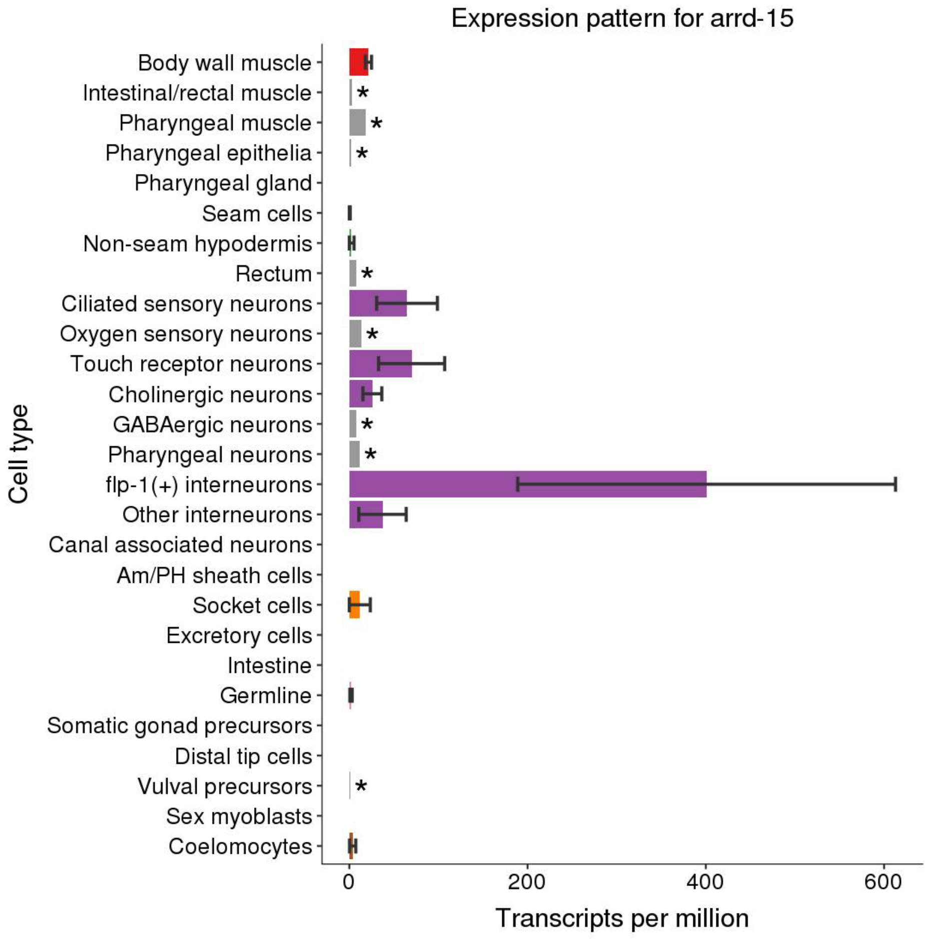

Figure S1. mRNA expression pattern of *sup-13/arrd-15.* Data are derived from GExplore 1.4 (http://genome.sfu.ca/gexplore/).

Table S1. *C. elegans* strains used in this study

Strain	Genotype	Reference
N2	Wild-type	Brenner (1974) [1]
RW2337	*sup-13(st210) III;unc-78(e1217) X*	Waterston (1988) [2]
CB1217	*unc-78(e1217) X*	Brenner (1974) [1]
ON5	*unc-78(gk27) X*	Ono (2001) [3]
ON320	*sup-13(st210) III;unc-78(gk27) X*	This study
ON341	*sup-13(st210) III*	This study
ON355	*aipl-1(kt4[aipl-1::3XFLAG::gfp]) V*	This study
ON362	*aipl-1(kt4[aipl-1::3XFLAG::gfp]) V;unc-78(gk27) X*	This study
ON357	*sup-13(st210) III; aipl-1(kt4[aipl-1::3XFLAG::gfp]) V;unc-78(gk27) X*	This study
ON381	*sup-13(st210) III; aipl-1(kt4[aipl-1::3XFLAG::gfp]) V*	This study
PHX4137	*arrd-15(syb4137[arrd-15::gfp]) III*	This study
ON369	*sup-13(st210) III;unc-78(gk27) X;ktEx252[myo-3p::gfp::ARRD-15a, pRF4]*	This study

[1]S. Brenner, The genetics of *Caenorhabditis elegans*. *Genetics* **77**, 71-94 (1974).

[2]R. H. Waterston, "Muscle" in The Nematode *C. elegans,* W. B. Wood, Ed. (Cold Spring Harbor Laboratory, 1988), pp. 281-335.

[3]S. Ono, The *Caenorhabditis elegans unc-78* gene encodes a homologue of actin-interacting protein 1 required for organized assembly of muscle actin filaments. *J Cell Biol* **152**, 1313-1319. (2001).
